# Effect of parasympathetic inhibition on expression of ILC2 cells in a mouse model of allergic rhinitis

**DOI:** 10.1016/j.waojou.2021.100582

**Published:** 2021-10-01

**Authors:** Binbin Hu, Yan Wang, Guotong Zheng, Hailin Zhang, Liyan Ni

**Affiliations:** a The Second Affiliated Hospital and Yuying Children's Hospital of Wenzhou Medical University, Wenzhou, 325027, China; b The Fifth People's Hospital of Shanghai, Fudan University, Shanghai, 200240, China; c Taizhou Women and Children's Hospital, Taizhou, 318000, China; d The Children's Hospital of Zhejiang University School of Medicine, Hangzhou, 310057, China

**Keywords:** Mouse model, Allergic rhinitis, Parasympathetic inhibition, ILC2s

## Abstract

**Background:**

We wanted to investigate whether parasympathetic inhibition affected the expression of type 2 innate lymphoid cells (ILC2s) in the nasal mucosa of a mouse model of allergic rhinitis (AR).

**Methods:**

Thirty male C57BL/6 mice were randomly divided into 3 groups: control group, AR group, AR-treated group. AR nasal symptoms were assessed on a semi-quantitative scale according to the frequencies of nose rubbing and sneezing and the degree of rhinorrhea. The expression of cytokines protein in serum was detected by enzyme linked immunosorbent assay (ELISA). The number of ILC2s in nasal mucosa was detected by immunofluorescence double staining assay. Quantitative real-time Polymerase Chain Reaction (qPCR) was used to detect the expression of ILC2-associated factor in nasal mucosa.

**Results:**

The symptom scores of the AR group were significantly higher than those of the control group and AR-treated group. The expression levels of mouse ovalbumin (OVA) specific IgE, IL4, IL5, and IL13 in the serum of AR group were significantly higher than those in the control group and AR-treated group. The number of ILC2s and the gene expression of ILC2s related factors GATA3, CD25 and CD90 (Thy1) in the nasal mucosa of the AR group were significantly higher than those of the control group and AR-treated group.

**Conclusions:**

We found that parasympathetic inhibition relieved AR symptoms and inhibited immune response of AR mice. ILC2s play an important role in the occurrence and development of AR, and parasympathetic nerve inhibition reduced the number of ILC2s and inhibited the cytokines expression by ILC2s. Our data provide information on the mechanism of action of parasympathetic inhibition in AR.

## Introduction

Allergic rhinitis (AR), a non-infectious chronic inflammatory disease, affects 10%–20% of the global population and the proportion is still increasing.^1^ The clinical symptoms of AR are usually paroxysmal sneezing, rhinorrhea, nasal congestion, and nasal itching, which have a serious impact on the quality of life of patients and consume huge social and medical costs. AR has become a research hot topic all over the world, but its pathogenesis has not been fully elucidated. The recognized pathogenesis is that when the nasal mucosa is exposed to allergens, dendritic cells (DCs) can uptake and present them to T helper 2 (Th2) cells. Th2 cells induce B cells to become allergen-specific IgE-producing plasma cells by synthesizing and secreting Th2-driving cytokines such as interleukin-13 (IL-13) and interleukin-4 (IL-4), and then producing specific IgE. Allergen-specific IgE antibodies attach to high-affinity IgE receptor FcεRI. Upon re-exposure, the allergen binds to the IgE on the surface of these cells and cross-links the IgE receptors, leading to activation of mast cells and basophils and the release of neuroactive and vasoactive mediators such as histamine and cysteinyl leukotrienes. Histamine has a direct effect on blood vessels (enhancing vascular permeability and plasma leakage) and sensory nerves, while leukotrienes are more likely to cause vasodilation. The excitement of the sensory nerve can cause nasal itching and sneezing, while the excitation of the parasympathetic nerve can stimulate glandular secretion and vasodilation, causing runny nose and other symptoms.^2^ It has been found that mast cells directly contacted and attached to the nerve through cell adhesion molecule 1 (CADM1),^3^^,^^4^ which kept increasing during inflammation of AR.^5^^,^^6^ And eosinophils are also found to accumulate around cholinergic nerves in allergic reactions.^7^ The mediators secreted by neurons, including neuropeptides and neurotransmitters, act on the homologous receptors of allergic immune cells (mast cells, dendritic cells, eosinophils, Th2 cells, and ILC2s) to drive or regulate immunity.^8^ In nasal mucosa, parasympathetic nerve has obvious functional advantages, while sympathetic nerve intervention has little effect on nasal mucosa inflammation.^9^ Moreover, it was found that ipratropium bromide, an anticholinergic drug, alleviated AR symptoms and reduced the number of eosinophils in nasal mucosa in a mouse model of AR.^10^ All these studies show that the parasympathetic nerve plays an indispensable role in the occurrence and development of AR.

Type 2 innate lymphoid cells (ILC2s) were recently reported to play a key role in the pathogenesis of allergic disease, sometimes more important than Th2 cells.^11^ They are derived from lymphoid progenitor and have functions similar to those of adaptive immune cells, but unlike lymphocytes of the adaptive immunity, ILC2s do not express antigen-specific receptors. Airway epithelial cells can secrete cytokines such as IL-25, IL-33, and thymic stromal lymphopoietin (TSLP) after being stimulated by allergens.^12^ Then ILC2s are stimulated to produce Th2 cytokines such as IL-4, IL-5, IL-9, and IL-13, which participate in Th2 immune response and are closely related to the occurrence and development of AR.^13^

Although some studies have verified that parasympathetic suppression can regulate the Th2 type immune response in the nasal mucosa of mice with AR, it is still not clarified whether parasympathetic suppression can affect the expression of ILC2s in the nasal mucosa of mice with AR. In this study, a mouse model of AR was established and treated with ipratropium bromide solution as a parasympathetic inhibitor. Nasal symptom scoring scale, immunofluorescence double staining, quantitative real-time PCR (qPCR), and enzyme-linked immunosorbent assay (ELISA) were used to investigate whether parasympathetic inhibition affected the expression of ILC2s in the nasal mucosa of the AR mouse model.

## Methods and materials

### Reagents

Ipratropium bromide was purchased from yuanye Bio-Technology Co., Ltd (Shanghai, China). Ovalbumin (OVA) and aluminum hydroxide were obtained from Sigma-Aldrich (St. Louis, MO, USA). Antibodies against CD90 (Thy1) (ab3105), ST2 (ab25877) were obtained from Abcam (Cambridge,UK). Secondary antibodies were obtained from Invitrogen (Carlsbad, CA, USA). IL-4, IL-5, IL-13 and mouse OVA specific IgE ELISA kits were purchased from Shanghai Xitang Biotechnology Co., Ltd (Shanghai, China). Trizol was obtained from Thermo Fisher scientific (Waltham, MA, USA). SYBR green was obtained from Bio-RAD Laboratories (Hercules, CA, USA).

### Establishment of AR mouse model

**Fig. 1:**
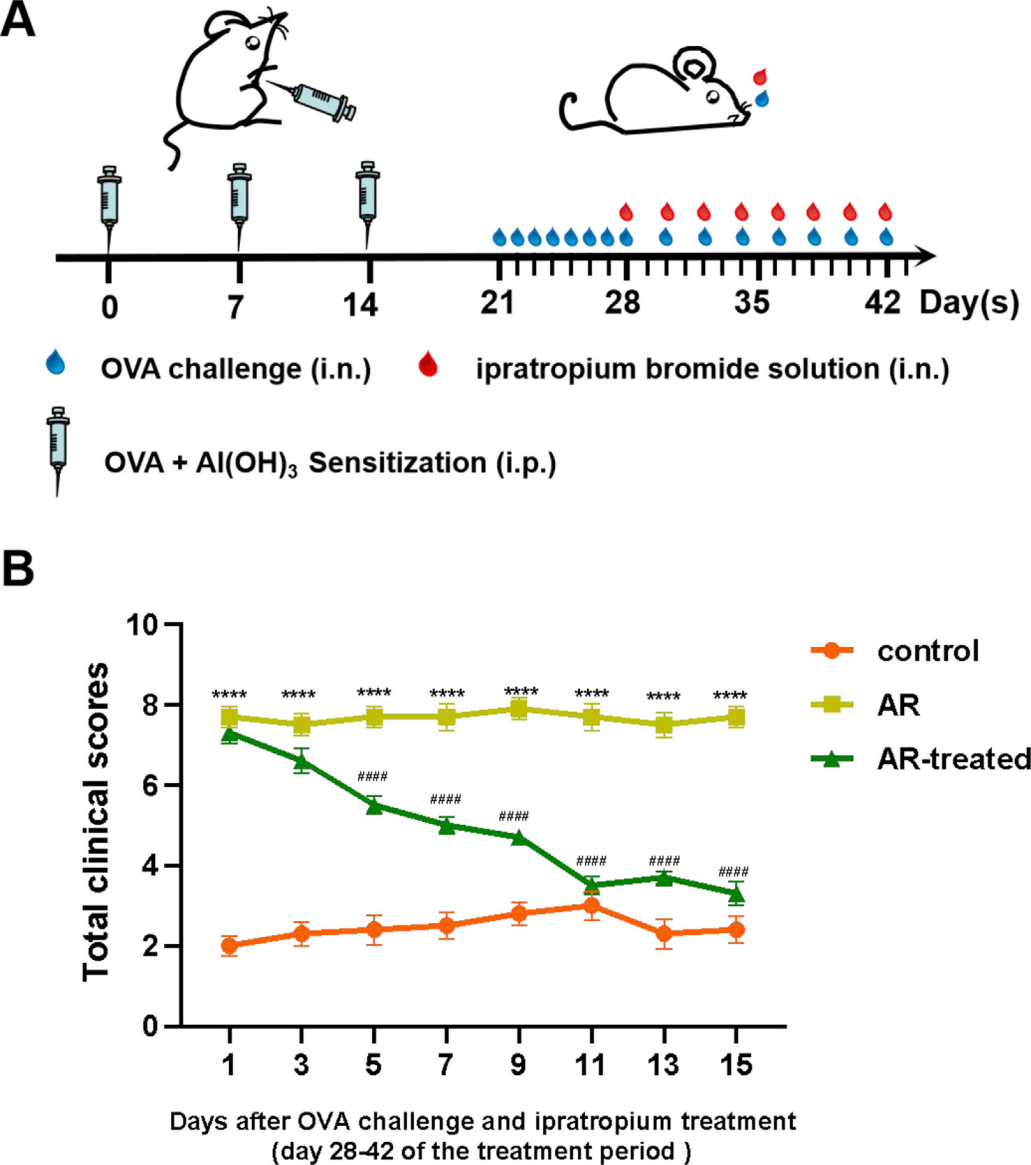
Establishment of AR model and symptom scores of three groups of mice. (A) On day 0, 7, and 14, the mice were sensitized by i.p. injection of OVA and aluminum hydroxide, followed by intranasal challenge with OVA once a day from day 21 to day 27. On every other day from day 28 to day 42, the mice in the AR group and the AR-treated group were treated with saline and ipratropium bromide respectively. The mice in the control group were injected with saline i.p. or i.n. according to the same schedule. All mice were sacrificed on day 43. (B) The mice were observed for 10 min after OVA challenge, and the nasal symptom scores of three groups were calculated. AR group vs Control group: ∗∗∗∗P < 0.0001. AR-treated group vs AR group: ^####^P < 0.0001. All data represent the mean ± SEM, n = 10

A total of 30 specific pathogen-free male C57BL/6 mice, aged 5 weeks, were purchased from Nanjing University Nanjing Biomedical Research Institute. The animal experiment in this study was approved by Animal Experimentation Ethics Committee. The 30 mice were randomly divided into 3 groups (n = 10 for each): control group, AR group, AR-treated group. The establishment of the AR model and the time of treatments are described below (Fig. 1A). In brief, each mouse in the AR group and AR-treated group was intraperitoneally (i.p.) injected with 0.2 mL suspension of OVA (0.125 mg/mL) and aluminum hydroxide (5 mg/mL) on day 0, 7, and 14. From day 21 to 27, AR group and AR-treated group were intranasally (i.n.) administrated with 20 μL OVA suspension (25 mg/mL). On day 27, the symptoms of each mouse were scored in 10 min after nasal challenge, and the scores higher than 5 indicated that the models were successfully established (Table 1).^14^ From day 28 to day 42, the AR group was i.n. stimulated with 20 μL OVA suspension (25 mg/mL) every other day. 20 μL ipratropium bromide solution (3 mg/mL) was instilled into the nasal cavity 15 min before the nasal challenge in the AR-treated group. The control group mice received saline i.p. or i.n. on the same schedule. Mice were sacrificed 24 h after the last stimulation, and the symptom scores and blood collection were completed before execution.

**Table 1:** Nasal symptom scoring in the AR mouse model

Score	Nasal symptoms		
	Number of sneezes per 20 min	Nasal mucus	Number of nasal rubbing per minute
0	None	None	None
1	1–3	Nostril	1–2
2	4–10	Outflow nostril	3–5
3	≥11	Flow to face	≥6

### ELISA

The expression of mouse OVA specific IgE, IL4, IL5, and IL13 were tested by employing an ELISA method. Briefly, 0.5 mL sample blood was collected from eye socket of each mouse. The blood was centrifuged at 4 °C and 1000 g for 10 min. The supernatant and pre-prepared standard solution were added into well plate. The immunosorbant assay was performed at 37 °C for 40 min. The absorbance value at 450 nm was measured by an enzyme labeling instrument. The concentration of mouse OVA specific IgE, IL4, IL5, and IL13 was calculated according to the standard curve.

### Immunofluorescence staining

Five mice from each group were randomly selected for immunofluorescence double staining to detect the number of ILC2s. The nasal mucosa wrapped by nasal bone was fixed in 4% neutral buffered formalin, then decalcified in EDTA solution. Three-micrometer slices were obtained and dewaxed in xylene, followed by ethanol gradient dehydration and washed in phosphate buffer solution (PBS). CD90 (Thy1) and ST2 antibodies (Abcam, Cambridge, UK) were employed to stain the aimed protein according to the manufacturer protocol. Photo capture was performed using upright fluorescence microscope (Zeiss, Germany).

### Quantitative real-time PCR (qPCR)

The nasal mucosa of other 5 mice in each group were used to detect the expression level of ILC2s associated factors by qPCR according to the manufacturer protocol. Total RNA from mouse nasal mucosa tissue was extracted and reverse transcription reaction was performed. As shown in Table 2, the CD25, CD90 (Thy1), GATA3, and GAPDH internal reference primer sequences were designed. Quantitative real-time PCR was performed using SYBR green on real-time PCR system. The relative value of miRNA levels was calculated by 2^−ΔΔCt^ method.^15^

**Table 2:** The prime sequence of real time fluorescent quantitative PCR

Prime	Sequence (5′-3′)	
	Forward	Reverse
CD25	5′-AGTTGTTTCTGTGGGTTG-3′	5′-CTGGCTAGTGAGGAATCTC-3′
CD90	5′-GGGCTCCTGTTTCTCCTT-3′	5′-TAGCCAACTTCACCACCA-3′
GATA3	5′-CCCATTAGCGTTCCTCCT-3′	5′-CCCTTATCAAGCCCAAGC-3′
GAPDH	5′-AGGTCGGTGTGAACGGATTTG-3′	5′-TGTAGACCATGTAGTTGAGGTCA-3′

### Statistical analysis

All the experimental data were analyzed by GraphpadPrism9 statistical software. Pairwise comparison was carried out by two-sample *t*-test, and one-way ANOVA was used in three groups. When 2 variables existed, two-way ANOVA analysis was performed, followed by Tukey's multiple comparison test. When P < 0.05, the difference was considered to be statistically significant.

## Results

### Evaluation of AR symptom scores in C57BL/6 mice

AR symptoms were scored according to the frequency of sneezing and wiping the nose and the degree of runny nose (Table 1). Compared with the control group, the AR group scored significantly higher at day 1, 3, 5, 7, 9, 11, 13, 15 (∗∗∗∗P < 0.0001), which was considered to successfully establish the AR model. Compared with the AR group, scores of the AR-treated group were significantly decreased after ipratropium bromide treatment, and significant differences occur at day 5, 7, 9, 11, 13, 15 (^####^P < 0.0001) (Fig. 1B).

### Detection of cytokines expression levels in mice serum by ELISA

**Fig. 2:**
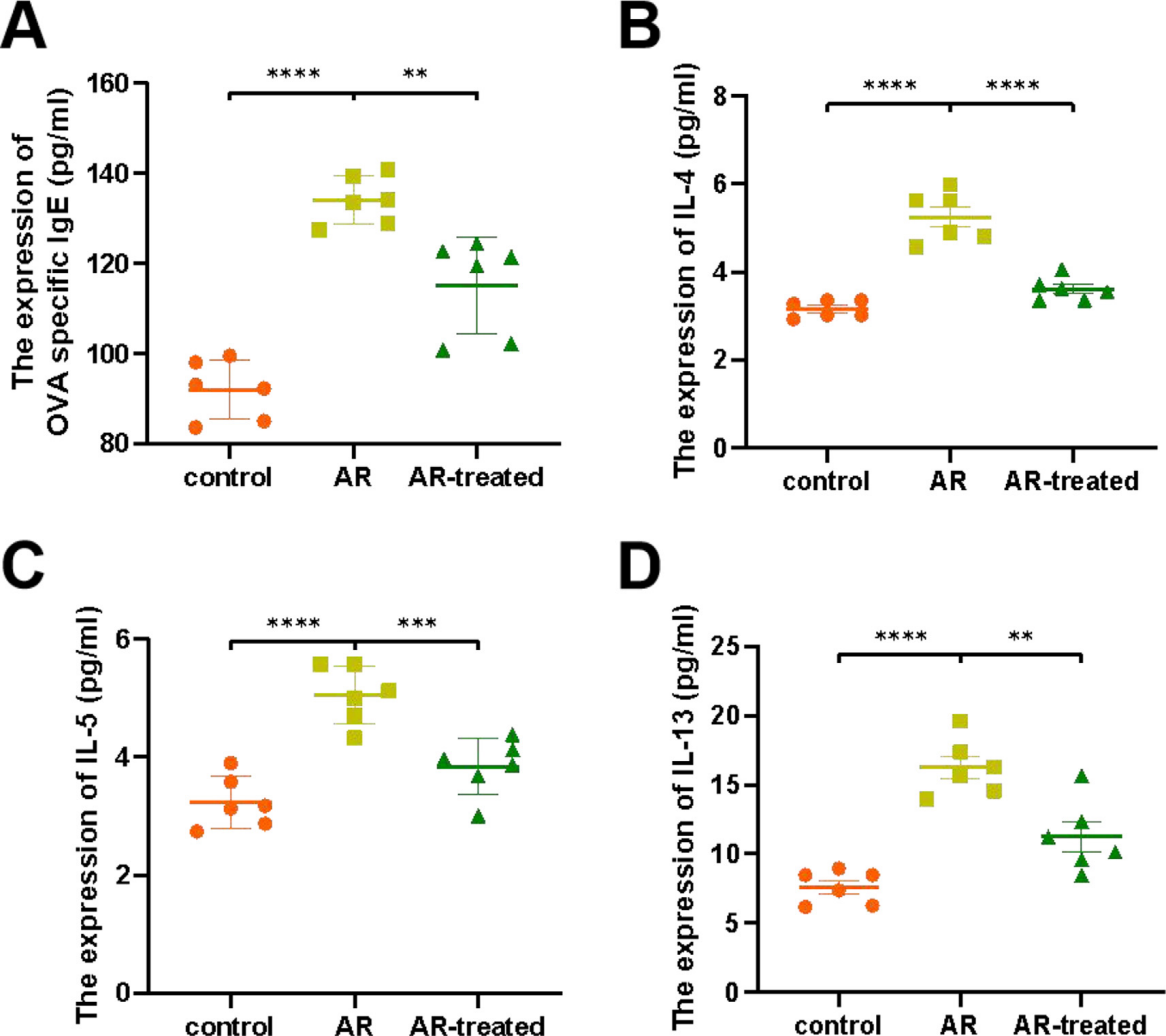
The expression levels of cytokines in mouse serum by ELISA. (A) The expression level of OVA specific IgE in mouse serum. (B) The expression level of IL-4 in mouse serum. (C) The expression level of IL-5 in mouse serum. (D) The expression level of IL-13 in mouse serum. All data represent the mean ± SEM, n = 6. ∗∗P < 0.01, ∗∗∗P < 0.001, ∗∗∗∗P < 0.0001

ELISA was used to detect the protein expression levels of IL-4, IL-5, IL-13, and mouse OVA specific IgE in mouse serum (Fig. 2). Compared with the control group, the serum levels of IL-4, IL-5, IL-13 and specific IgE of the AR group were significantly increased, and the difference was statistically significant. Compared with the AR group, the serum levels of IL-4, IL-5, IL-13, and specific IgE of the AR-treated group were significantly decreased, and the difference was statistically significant.

### Detection of the number of ILC2s in nasal mucosa tissues of mice by immunofluorescence double staining test

**Fig. 3:**
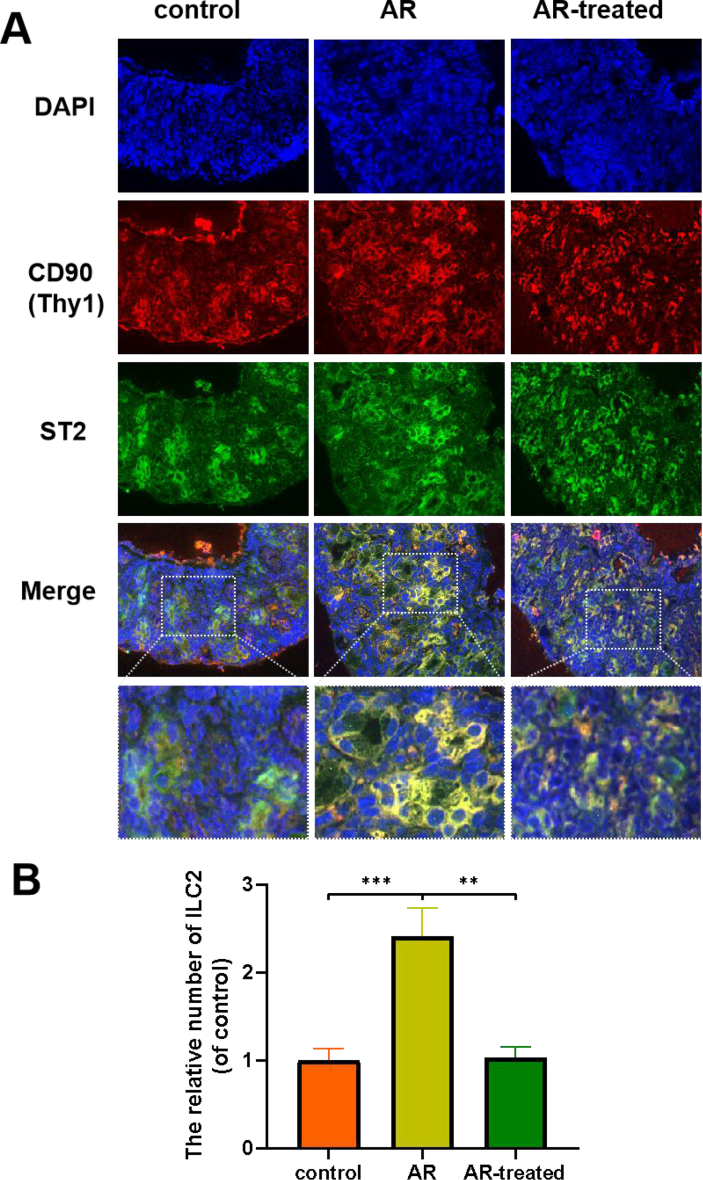
Observation of ILC2s in nasal mucosa of mice by immunofluorescence double staining test. (A) Representative immunofluorescence double stained sections of nasal mucosa from each group (×400). (B) Analysis of the relative number of double positive cells. The data are presented as the mean ± SEM, n = 5. Control group vs AR group: ∗∗∗P < 0.001. AR group vs AR-treated group: ∗∗P < 0.01

Double immunofluorescence staining for CD90 (Thy1) and ST2 showed that the number of double positive cells in the AR group was significantly higher than that in the control group. In contrast, the immunoreactivity of CD90 (Thy1) and ST2 was greatly attenuated after the treatment of ipratropium bromide (Fig. 3A). We calculated the relative number of double positive cells, the difference was statistically significant (Fig. 3B).

### Detection of mRNA expression levels of ILC2s associated factors in mice nasal mucosa

**Fig. 4:**
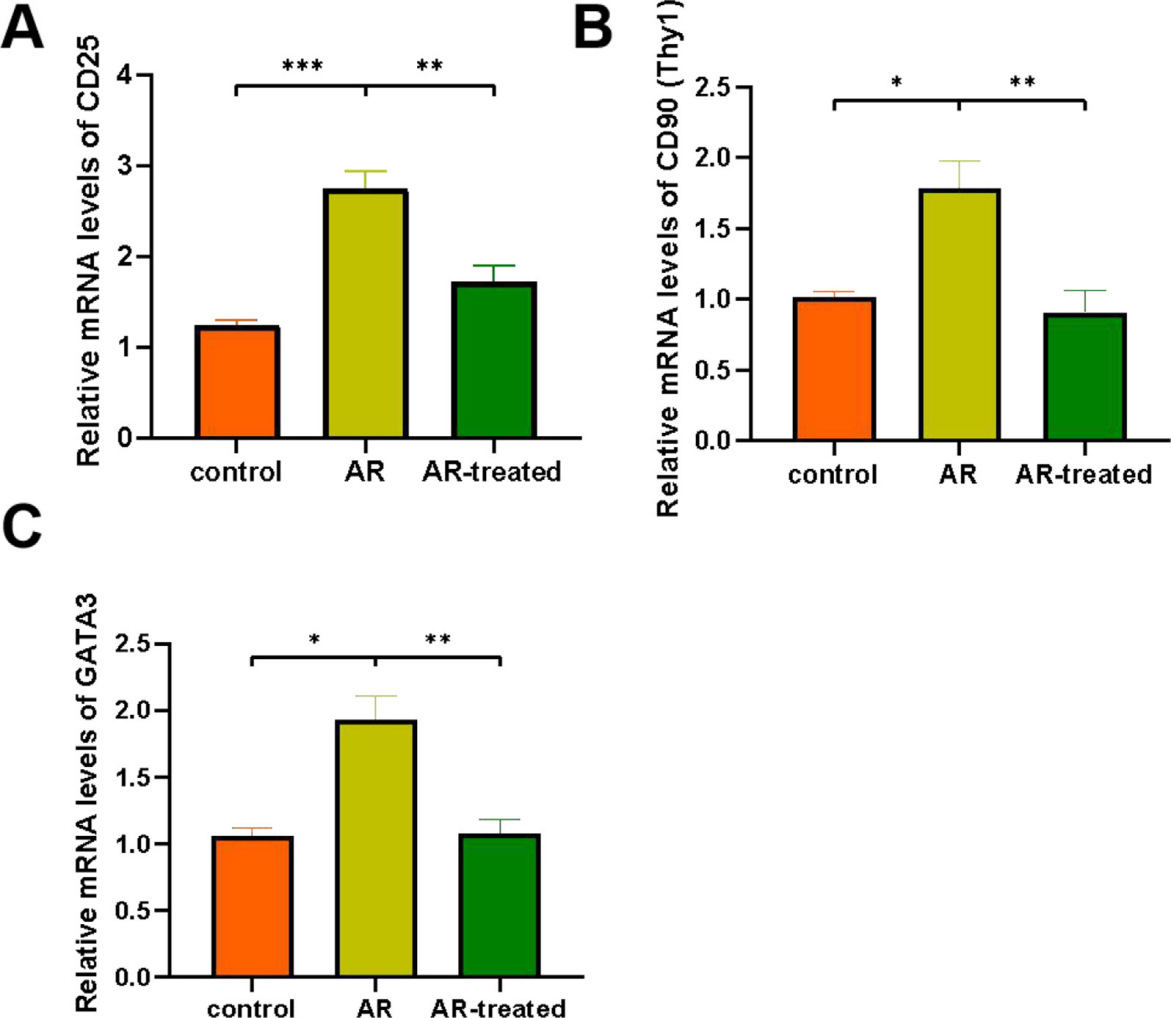
The expression levels of mRNA in mice nasal mucosa by quantitative real-time PCR. (A) The expression level of CD25 in nasal mucosa. (B) The expression level of CD90 (Thy1) in nasal mucosa. (C) The expression level of GATA3 in nasal mucosa. All data represent the mean ± SEM, n = 5. ∗P < 0.05, ∗∗P < 0.01, ∗∗∗P < 0.001

The mRNA expression levels of CD25, CD90 (Thy1), GATA3 in mice nasal mucosa were detected by quantitative real-time PCR. Compared with the mice in control group, the expression of CD25, CD90 (Thy1), GATA3 in the nasal mucosa of the mice in AR group was significantly increased in mRNA level, and the difference was statistically significant. Compared with the mice in AR group, the expression of CD25, CD90 (Thy1), GATA3 in the nasal mucosa of the mice in AR-treated group was significantly decreased in mRNA level, and the difference was statistically significant (Fig. 4).

## Discussion

AR is one of the most common diseases in otorhinolaryngology, head and neck surgery. But its pathogenesis remains to be clarified, and the clinical symptoms of some patients can not be effectively relieved. Therefore, there is an urgent need to further study the mechanism of AR. In recent years, the study of ILC2s, innate lymphocytes that can participate in adaptive immunity, provides a new research scope for the pathogenesis of AR. ILC2s were first discovered in 2001, Fort et al^16^ stimulated T and B cell deficient mice with IL-25, and it was found that Th2 cytokines such as IL-4, IL-5 and IL-13 were still produced in mice, indicating that there were innate immune cells in mice participating in Th2 immune response. In 2010, 3 research groups^17^, ^18^, ^19^ described this type of cells that produced Th2 cytokines respectively, and reached a consensus and named them ILC2s. Airway epithelial cells release cytokines such as IL-25, IL-33, and TSLP to activate ILC2s. ILC2s synthesize and secrete Th2 cytokines (IL-4, IL-5, IL-9, and IL-13), which promote eosinophils and mast cells to participate in allergic reactions.^20^ ILC2s can express CD90 (Thy1), CD127 (IL7Rα), KLRG1, ICOS, ST2 (IL33R), and CD25 (IL-2Rα). Their expression levels vary among tissues, but show quite characteristic signatures of transcriptome in comparison with that in ILC1 and ILC3.^21^^,^^22^ Allergens such as ovalbumin and dust mites were used to sensitize and stimulate BALB/c mice to establish respiratory allergic inflammation mainly caused by Th2 mediated eosinophilic infiltration.^23^, ^24^, ^25^, ^26^ BALB/c mice immune responses tend to be Th2 response and are widely used for AR mice model.^27^ However, C57BL/6 mice can be used as ILC2s mediated allergic inflammation, which reduce the influence of Th2 type adaptive immunity and better reflect the important role of innate immunity in respiratory allergic inflammation.^28^

Although AR is an immune disease, neuroregulation plays a very important role in occurrence and development of AR. In recent years, some research groups proposed that nerve cells and immune cells can interfered with each other in specific anatomical sites to form a neuroimmune cell unit (NICU) to play a regulatory role.^29^ In 2013, the Locksley^30^ team reported that biorhythm and diet could regulate the secretion of vasoactive intestinal peptide (VIP). VIP can bind to VIP receptor 2 (VPAC2) on the surface of ILC2s, thus promoting ILC2s to secrete IL-5, which can recruit eosinophils, participate in infection and immune response, and sensory neurons can secrete vasoactive intestinal peptide under IL-5 stimulation. This forms a positive feedback loop in allergic diseases.^31^ In 2017, 3 research groups^32^, ^33^, ^34^ simultaneously proposed that neuropeptide neuromedin U (NMU) produced by cholinergic neurons were with the ability to activate mouse ILC2s in C57BL/6 mice. ILC2s can express NMU receptor Nmur1 in mouse lungs and small intestine, but not in other lymphocytes and myeloid cells.

However, few studies on whether parasympathetic inhibition affects the expression of ILC2s in AR mice. In this study, we established a C57BL/6 mouse model of AR and treated the mice with parasympathetic inhibitor ipratropium bromide to explore whether parasympathetic inhibition affects the expression of ILC2s in nasal mucosa of AR mice. The ILC2s were defined as Lin-ST2^+^CD45^+^CD127^+^KLRG1^+^Thy-1^+^ cells.^15^ CD90 (Thy1) and ST2 are important cell surface markers related to ILC2s. CD90 (Thy1) and ST2 immunofluorescence double staining detected the number of ILC2s in nasal mucosa tissue.^35^ The results showed that the number of ILC2s in nasal mucosa of ipratropium bromide group was significantly lower than that of AR group, indicating that parasympathetic nerve inhibition could reduce the number of ILC2s in nasal mucosa of AR mice. GATA3 is a very important transcription factor in the differentiation and development of ILC2s. It can inhibit the differentiation of common lymphoid progenitor cells into B cells, thus promoting the differentiation of T and ILC cells.^36^, ^37^, ^38^ GATA3 can regulate the expression of many key genes in ILC2s, and the lack of GATA3 will lead to the inability of ILC2s to secrete IL-5 and IL-13.^39^^,^^40^ CD25 and CD90 (Thy1) are receptors expressed on the surface of ILC2s after maturation, and they are the surface markers of ILC2s. Though GATA3, CD25 or CD90 (Thy1) in nasal mucosa is not limited to ILC2s, these factors belong to ILC2s related factors. So we detected these factors to observe the function of ILC2s and the immunity of AR mice to some extent. In this study, it was found that the gene expression levels of GATA3, CD25 and CD90 (Thy1) in AR-treated group were lower than those in the AR group. ILC2s produce and secrete Th2 cytokines IL-4, IL-5 and IL-13, which play a very important role in AR. Among the 3 cytokines, IL-4 can promote the synthesis and release of IgE. IL-5 is very important for eosinophil homeostasis and B cell function. IL-13 can promote the secretion of mucous cells and cause goblet cell proliferation. In this study, the expression levels of specific IgE, IL-4, IL-5, and IL-13 in serum of mice in each group were detected by ELISA. The results showed that ipratropium bromide could significantly reduce the expression levels of specific IgE, IL-4, IL-5, and IL-13 in serum of AR model mice. The expression of IL-4, IL-5, and IL-13, in some degree, indicated that parasympathetic inhibition could inhibit the function of ILC2s and these cytokines expression also indicated that parasympathetic inhibition could inhibit immune response of mice with AR. In addition, we also found that ipratropium bromide, a parasympathetic inhibitor, could significantly improve the symptoms of AR in AR model mice, which was consistent with findings from Li.^10^

To sum up, our experimental study confirmed that ILC2s play an important role in the occurrence and development of AR. Ipratropium bromide, an inhibitor of parasympathetic nerve, relieved the symptoms of AR, inhibited the number and function of ILC2s and immunity of mice with AR, which provided a new therapeutic basis for the treatment of AR by parasympathetic inhibition, but the specific mechanism of parasympathetic inhibition on ILC2s still needs to be further explored.

## Abbreviations

AR, allergic rhinitis; ILC2s, Type 2 innate lymphoid cells; OVA, ovalbumin; DC, dendritic cells; IL, interleukin; TSLP, thymic stromal lymphopoietin; qPCR, Quantitative real-time Polymerase Chain Reaction; TH, helper T cell; CADM1, cell adhesion molecule 1; PBS, phosphate buffered saline; IgE, immunoglobulin E; ELISA, enzyme linked immunosorbent assay; VIP, vasoactive intestinal peptide; NMU, neuropeptide neuromedin U

## Ethics statement

The animals used in this study were approved by the Animal Experimentation Ethics Committee of Wenzhou Medical University, Wenzhou, China. The living condition and experimental procedures were conducted in accordance with the National Institutes of Health Guideline concerning the Care and Use of Laboratory Animals.

## Availability of data and materials

The datasets used and/or analyzed during the current study are available from the corresponding author on reasonable request.

## Author contributions

Liyan Ni and Hailin Zhang conceptualized and designed the study, and approved the final manuscript as submitted. Binbin Hu, Yan Wang, Guotong Zheng collected the sample, performed the experiment, data collection and statistics, drafted the initial manuscript, reviewed and revised the manuscript, and approved the final manuscript as submitted.

## Consent for publication

All authors agreed to publication of the work.

## Funding

This work was supported by grants from the Science and Technology Plan Project of Taizhou (1902ky75), 10.13039/501100007194Wenzhou Science and Technology Bureau (Y20190154), the Special Project for Significant New Drug Research and Development in the Major National Science and Technology Projects of China (2020ZX09201002), 10.13039/501100001809National Natural Science Foundation of China (81973382).

## Declaration of competing interest

The authors declare that they have no relevant conflicts of interest.
